# 

**Published:** 1900-09-15

**Authors:** 


					THE HOSPITAL. "The standard of highest purity."?Lancet. September i5th, 1900.
CADBURY'S U CADBURY'S
COGOA ^ COCOA
ABSOLUTORY POU. NO DRUGS OR ALKALIES USED.
Western Infirmary
io. 729. Vol. XXVIII. [JSftSiS SiTUKDAT'
September 15, 1900.
[-SubBcriptionT^ms,] pRICB TWOPENCE.

				

